# Poly-Thymidine Oligonucleotides Mediate Activation of Murine Glial Cells Primarily Through TLR7, Not TLR8

**DOI:** 10.1371/journal.pone.0022454

**Published:** 2011-07-21

**Authors:** Min Du, Niranjan B. Butchi, Tyson Woods, Karin E. Peterson

**Affiliations:** Laboratory of Persistent Viral Diseases, Rocky Mountain Laboratories, National Institute of Allergy and Infectious Diseases, Hamilton, Montana, United States of America; Indian Institute of Science, India

## Abstract

The functional role of murine TLR8 in the inflammatory response of the central nervous system (CNS) remains unclear. Murine TLR8 does not appear to respond to human TLR7/8 agonists, due to a five amino acid deletion in the ectodomain. However, recent studies have suggested that murine TLR8 may be stimulated by alternate ligands, which include vaccinia virus DNA, phosphothioate oligodeoxynucleotides (ODNs) or the combination of phosphothioate poly-thymidine oligonucleotides (pT-ODNs) with TLR7/8 agonists. In the current study, we analyzed the ability of pT-ODNs to induce activation of murine glial cells in the presence or absence of TLR7/8 agonists. We found that TLR7/8 agonists induced the expression of glial cell activation markers and induced the production of multiple proinflammatory cytokines and chemokines in mixed glial cultures. In contrast, pT-ODNs alone induced only low level expression of two cytokines, CCL2 and CXCL10. The combination of pT-ODNs along with TLR7/8 agonists induced a synergistic response with substantially higher levels of proinflammatory cytokines and chemokines compared to CL075. This enhancement was not due to cellular uptake of the agonist, indicating that the pT-ODN enhancement of cytokine responses was due to effects on an intracellular process. Interestingly, this response was also not due to synergistic stimulation of both TLR7 and TLR8, as the loss of TLR7 abolished the activation of glial cells and cytokine production. Thus, pT-ODNs act in synergy with TLR7/8 agonists to induce strong TLR7-dependent cytokine production in glial cells, suggesting that the combination of pT-ODNs with TLR7 agonists may be a useful mechanism to induce pronounced glial activation in the CNS.

## Introduction

Neuroinflammation, including cytokine/chemokine production by resident glial cells, is a common response to various types of central nervous system (CNS) insults, including viral infections [1]–[5]. Understanding the events that trigger the initiation of neuroinflammatory responses is important in determining how viruses induce damage to the CNS. The host recognizes viral infections through the detection of pathogen-associated molecular patterns (PAMPs), repeated structural motifs generated by microbes that are not normally found in the host [6], [7]. These PAMPs are recognized by pattern recognition receptors (PRRs) expressed by multiple cell types including dendritic cells and macrophages. Activation of these cells via PRRs promotes rapid inflammatory and anti-microbial responses [6]. Two PRRs that are important for the recognition of viruses are toll-like receptor 7 (TLR7) and TLR8. These two receptors are closely related endosomal TLRs that recognize guanosine and uridine-rich viral ssRNA, including RNA from virus families that are known to infect the CNS and induce neurological disease [8]–[11]. TLR7 and TLR8 can also be stimulated by synthetic molecules like imidazoquinoline compounds and guanosine analogs, which are currently used as anti-viral therapeutics [12]–[14].

The function of TLR7 and TLR8 in activation of dendritic cells in the periphery is well described [8], [9], [11], [15], [16]. However, the role of these receptors in the CNS immune response is still under investigation. In the brain, TLR7 is readily detected on ependymal cells and brain capillary endothelia [17], [18]. Following infection, TLR7 expression can also be detected on a number of cell types including astrocytes, microglia, endothelia and cerebellar granular neurons [18]. TLR7 can contribute to innate immune responses in the CNS as demonstrated by both agonist and viral infection studies [17], [19]–[22].

The impact of TLR8 in the CNS is not as clear, particularly in mouse models of virus infection. Although TLR8 is functional in humans, several studies using TLR7-deficient mice have indicated that TLR8 is not functional in mice [9], [11], [13]. Murine TLR8 contains a five amino acid deletion in the ectodomain, which appears to be required for ligand recognition, but not for dimerization or intracellular localization [23]. However, recent studies have suggested that TLR8 may be functional in mice through the recognition of an alternative ligand. Vaccinia virus DNA or synthetic phosphothioate poly-adenosine (pA) or poly-thymidine (pT) oligonucleotides (ODNs) were shown to stimulate murine cells via TLR8 [24]. Murine TLR8-transfected human embryonic kidney-293 (HEK-293) cells were activated when stimulated with a combination of TLR7/8 agonist CL075 (3M002) and pT-ODNs [25]. Thus, pT-ODNs either alone or in combination with TLR7/8 agonists may provide a mechanism to study the activation of murine TLR8 in the CNS.

In the current study, we analyzed the ability of pT-ODNs, either independently or in combination with CL075, to induce activation of glial cells. We found that pT-ODNs alone did not induce significant glial activation. Interestingly, the combination of pT-ODNs with CL075 induced a substantially heightened cytokine response compared to CL075 alone, but did not alter expression of other glial activation markers. TLR8, along with TLR7, was readily detected on both primary microglia and astrocytes. However, studies with TLR7-deficient mice demonstrated the glial activation and cytokine induction associated with either CL075 or pT-ODN/CL075 stimulation was dependent on TLR7. Therefore, although TLR8 is expressed on murine microglia and astrocytes, it appears to only have a minor influence on the innate immune responses of glial cells to either conventional or alternative ligands. This study differs from previous studies with TLR8-transfected HEK cells and suggests that basal levels of murine TLR8 expression may not be sufficient for cellular activation via conventional or alternative TLR8 ligands. Furthermore, it demonstrates that alternative TLR8 ligands such as pT-ODNs can also enhance TLR7-mediated responses, independently of TLR8.

## Results

### TLR8 expression on mixed cortical cells

**Figure 1:**
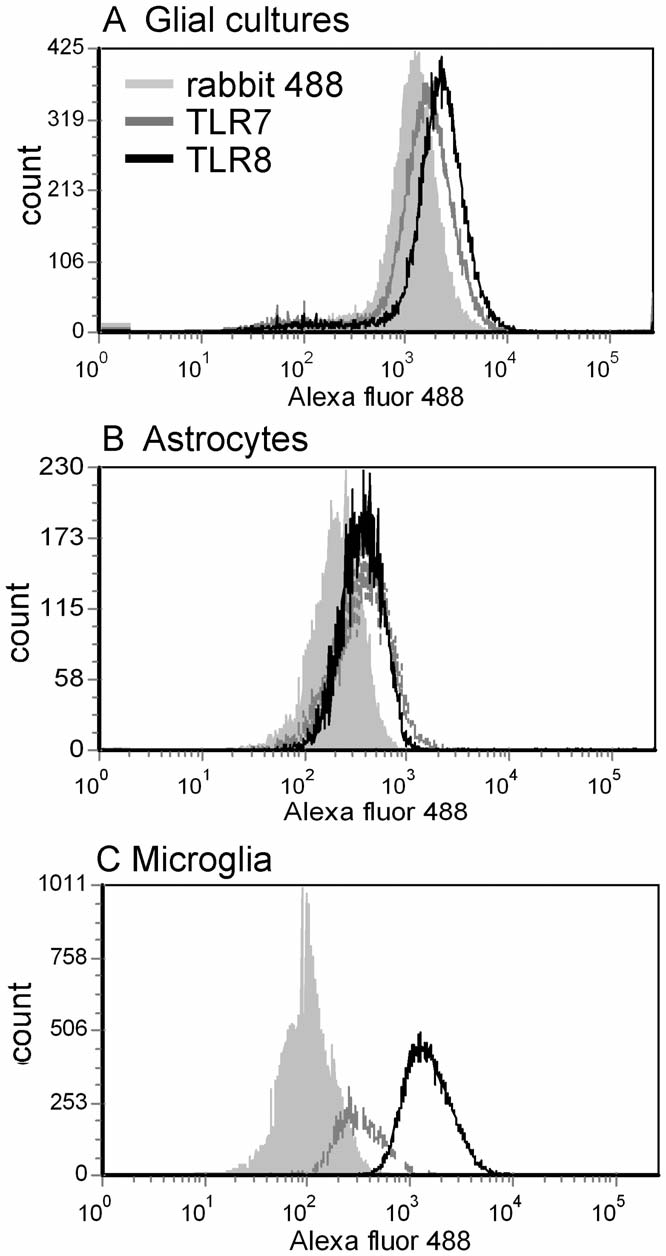
TLR7 and TLR8 protein expression on (A) primary mixed cortical cells, (B) purified astrocytes and (C) purified microglia. Primary cortical cells were generated as described in the materials and methods. Cells were either cultured as a mixed cell population or used to generate >95% pure microglia or astrocyte cultures. At 7–10 days post culture, cells were analyzed for TLR7 or TLR8 expression by flow cytometry using rabbit anti-TLR7 (gray line) or goat anti-TLR8 antibodies (black line). Data are shown in comparison to secondary alone antibody (anti-rabbit 488, gray filled). Isotype controls and rabbit anti-goat 488 antibodies were comparable to goat anti-rabbit staining (data not shown). Data are representative of at least two replicate experiments for each cell population.

Neither TLR7 nor TLR8 is readily detected on glial cells by immunohistochemistry staining of brain tissue [18]. However, glial cells do respond to TLR7/ TLR8 agonist stimulation *in vivo* indicating that they do express TLR7 and/or TLR8 [19]. Analysis of primary cortical cultures composed primarily of astrocytes but also containing microglia, demonstrated the expression of both TLR7 and TLR8 (Fig. 1A). Analysis of purified astrocytes or microglia also indicated TLR7 and TLR8 expression by both cell types, although on microglia TLR8 was expressed at higher levels than TLR7 (Fig. 1B, C).

### Glial Cell Activation Following CL075/pT-ODN Stimulation

**Figure 2:**
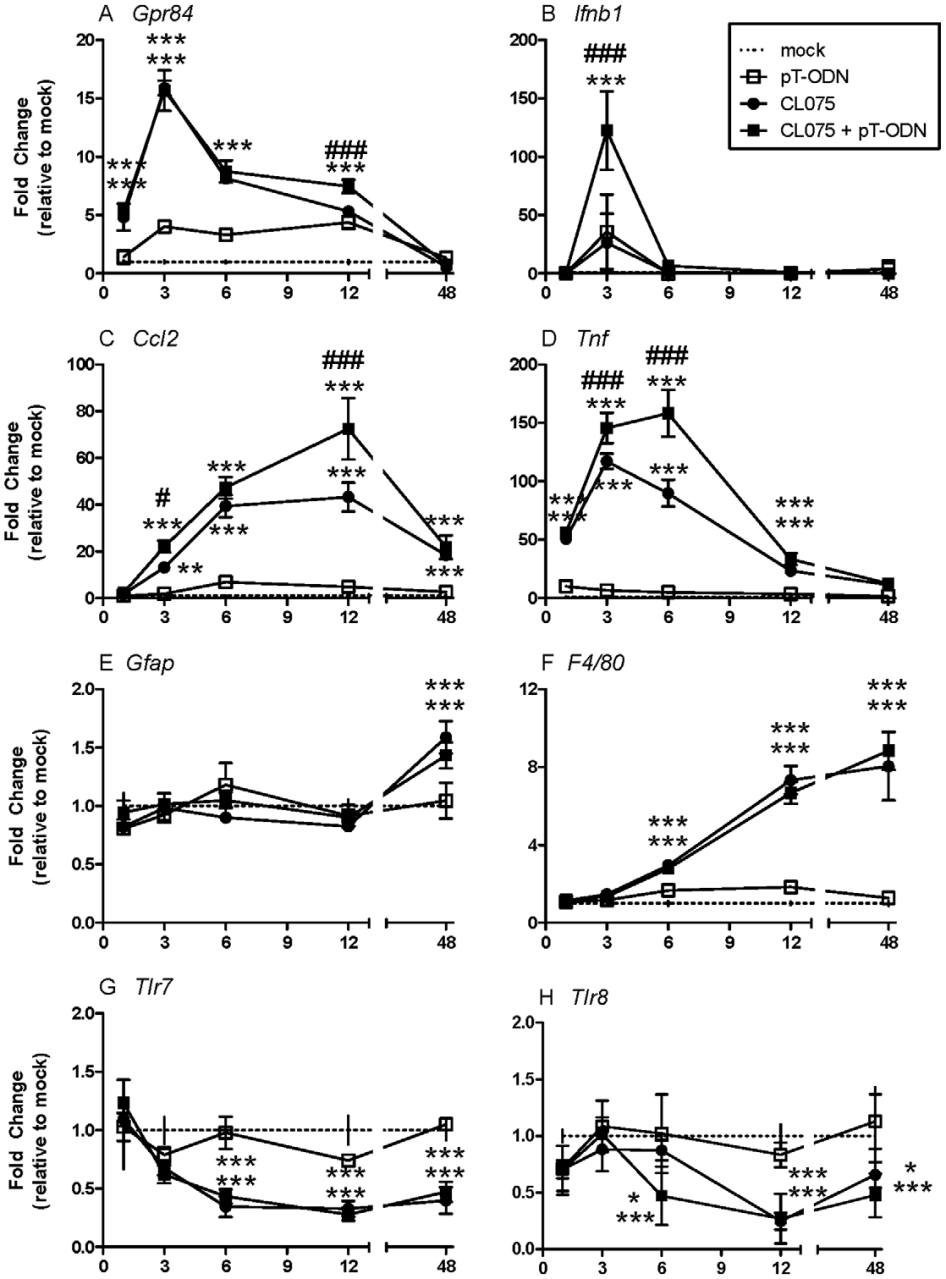
mRNA expression by primary cortical cells following pT-ODN/CL075 co-stimulation. Primary cortical cultures were generated as described in materials and methods. Glial cells were stimulated with 20 µM of CL075 and/or 1 µM pT-ODN. RNA was isolated from cells at indicated times post stimulation. RNA samples were processed for real-time quantitative RT-PCR analysis. Data were calculated relative to the expression of *Gapdh* mRNA and then compared as fold change relative to the average of mock infected controls. Data are the mean ± SEM of three wells per group and are representative of two replicate experiments. Statistical analysis was completed by two-way ANOVA with Newman-Keuls post test. Asterisks (*) directly above data symbols represent a statistically significant difference compared to media alone controls. Number sign (#) above data symbols represent a significant difference between CL075 and CL075/pT-ODN stimulated groups. * or # P<0.05, ** or ## P<0.01, and *** or ### P<0.001.

Time course analysis of mixed cortical cells stimulated with the TLR7/8 agonist CL075 showed four primary profiles of gene expression. mRNA expression of G-protein coupled receptor 84 (*Gpr84*) and type I interferon beta (*Ifnb1*) peaked within 3 hours post stimulation (hps) (Fig. 2A, B), while mRNA expression of proinflammatory cytokines such as *Ccl2* and *Tnf* peaked within 6–12 hps (Fig. 2C, D). mRNA for the microglia activation marker *F4/80* was upregulated at later time points with peak expression at or after 48 hps (Fig. 2F). The astrocyte activation marker glial fibrillary acidic protein (*Gfap*) was also upregulated in this time frame; however, this low increase was below the detection limit (one cycle, 2 fold) for real-time PCR (Fig. 2E). In contrast, *Tlr7* and *Tlr8* mRNA levels were downregulated following CL075 stimulation (Fig. 2G, F), which is similar to what is observed in bone marrow derived dendritic cells following TLR7 stimulation [26].

In comparison to CL075 stimulation, stimulation with pT-ODNs alone failed to induce expression of proinflammatory cytokines or glial activation markers (Fig. 2). The only detectable increase in cytokine mRNA production following pT-ODN stimulation alone was *Ifnb1* mRNA, although this was not statistically significant (Fig. 2B). Dose curve analysis using 5–10 fold lower or higher concentrations of pT-ODNs also failed to induce significant cytokine mRNA production (data not shown).

To examine whether pT-ODNs would alter the cytokine response to TLR7/8 agonists, cells were stimulated with 1 µM of pT-ODNs in combination with CL075. The costimulation with pT-ODNs induced a substantial increase in cytokine mRNA expression in glial cells compared to CL075 alone (Fig. 2B–D). Interestingly, the combined agonists did not enhance expression of glial activation markers *Gfap* or *F4/80* and did not affect *Tlr7* or *Tlr8* mRNA expression (Fig 2E–H).

**Figure 3:**
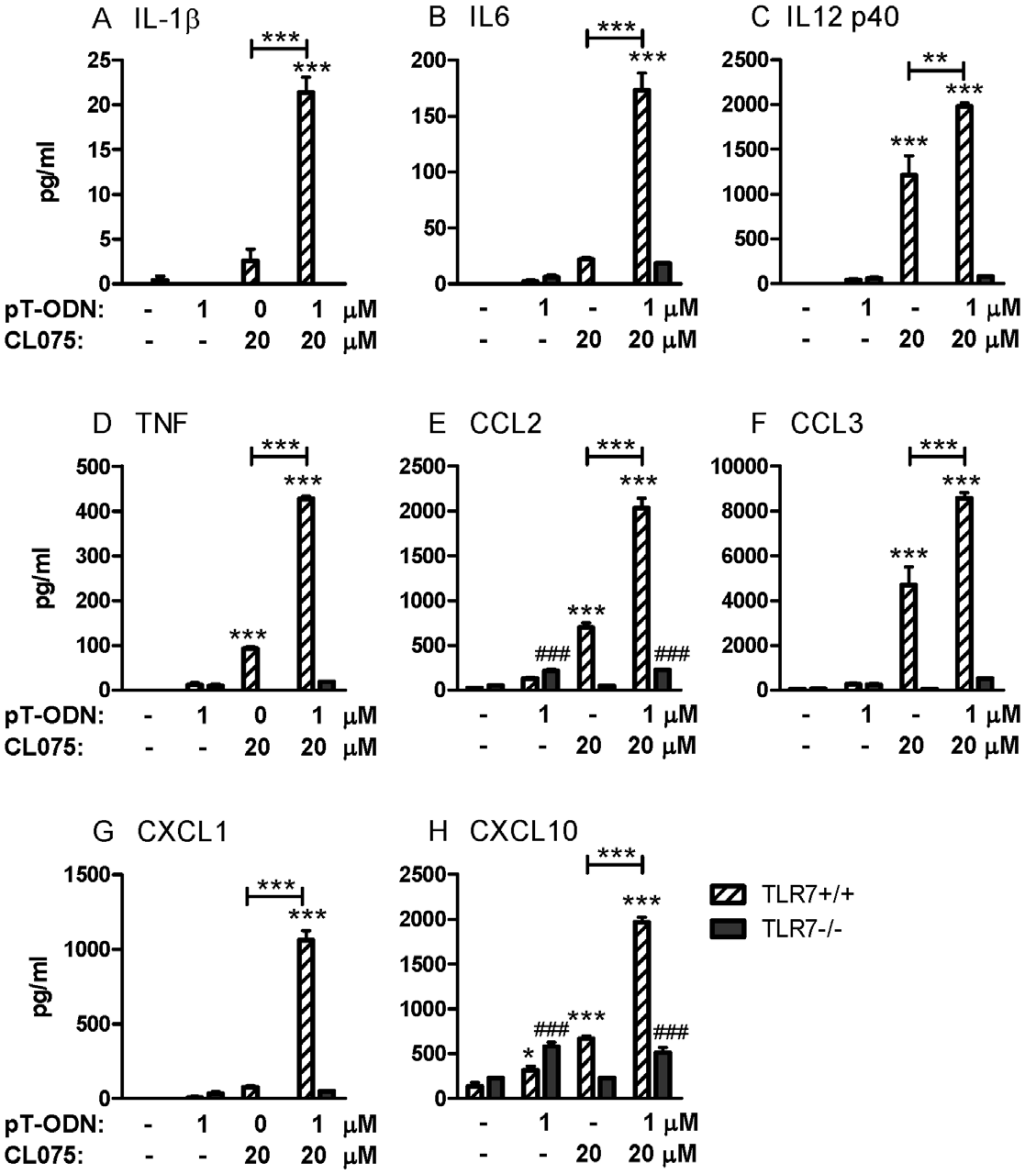
CL075/pT-ODN-induced production of (A–D) proinflammatory cytokines and (E–H) chemokines by primary cortical cells is dependent on TLR7. Primary cortical cultures from wildtype or TLR7-deficient mice were stimulated with 20 µM CL075 and/or 1 µM pT-ODN. Supernatants were collected at 48 hps and analyzed for cytokine and chemokine levels using a cytokine 20-plex multi-plex bead array. Data were calculated as pg/ml using in-plate standard curves. Data are the mean ± SEM of three wells per group and are representative of two replicate experiments. Statistical analysis was completed by One-way ANOVA with a Newman-Keuls post test within each group. Symbols above columns indicate a statistically significant difference compared to media controls. * P<0.05, ** P<0.01, and *** P<0.001 for wildtype mice; # P<0.05, ## P<0.01, and ### P<0.001 for TLR7-deficient mice. Symbols above lines between two columns indicate a statistically significant difference between the two indicated groups.

Analysis of cytokine protein levels in supernatants from stimulated cells demonstrated a similar response, with little to no cytokine expression following pT-ODN stimulation with the exception of low levels of CCL2 and CXCL10 (Fig. 3E, H). In contrast, significant production of cytokines was observed in supernatants following CL075 stimulation and a synergistic effect on cytokine production was observed in cells stimulated with both pT-ODNs and CL075 (Fig. 3). Similar results were observed with 5 µM of pT-ODNs in combination with 20 or 100 µM of CL075 (data not shown). Thus, although pT-ODNs did not induce glial stimulation by themselves, they did enhance the cytokine response induced by CL075.

**Figure 4:**
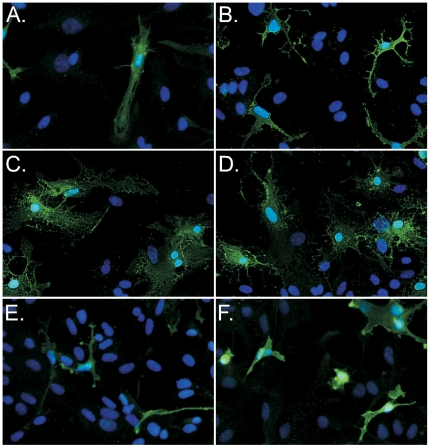
CL075, but not pT-ODN induces changes in microglia morphology. Primary cortical cultures from wildtype mice were stimulated with 20 µM CL075 and/or 1 µM pT-ODN. After 48 hrs, cells were fixed and then stained for the microglia/macrophage marker, Iba1 using an Alexafluor-488 conjugated anti-rabbit antibody for detection. Iba1 positive cells in (A) mock (unstimulated) and (B) pT-ODN-stimulated cultures were primarily elongated with only a few amoeboid cells. In contrast, Iba1 positive cells in (C) CL075 or (D) CL075/pT-ODN stimulated cultures were completely amoeboid in appearance. However, Iba1 positive cells from (E–F) TLR7-deficient mice did not take on the amoeboid appearance after stimulation with (E) CL075 or (F) CL075/pT-ODN. Images are representative of cells in each well and are the results of one of two replicate experiments.

Microglia often alter their morphology following activation. Iba1 positive microglia cells in primary cultures demonstrated a shift in morphology towards an amoeboid-like phenotype following CL075 stimulation, but not pT-ODN stimulation (Fig. 4A–C). CL075/pT-ODN stimulated cells had similar morphology to CL075 stimulated cells (Fig. 4D). This indicates that CL075, but not pT-ODNs were capable of activating primary microglia cells.

### pT-ODN/CL075-induction of cytokines is TLR7 dependent

Previous studies demonstrated that addition of pT-ODNs enhanced cytokine responses induced by CL075 in murine TLR8-transfected HEK cells, but not TLR7-transfected HEK cells [25]. To examine whether TLR7 mediated the pT-ODN/CL075- induced cytokine production, cortical cells from TLR7-deficient mice were stimulated with pT-ODNs with or without CL075. Surprisingly, the absence of TLR7 ablated the majority of the cytokine/chemokine response to pT-ODN/CL075 stimulation, including the induction of type I IFN responses (Fig. 3, 5A). Thus, in murine glial cells, the ability of pT-ODN to provide costimulation to TLR7/8 agonists was mediated via TLR7 stimulation.

**Figure 5:**
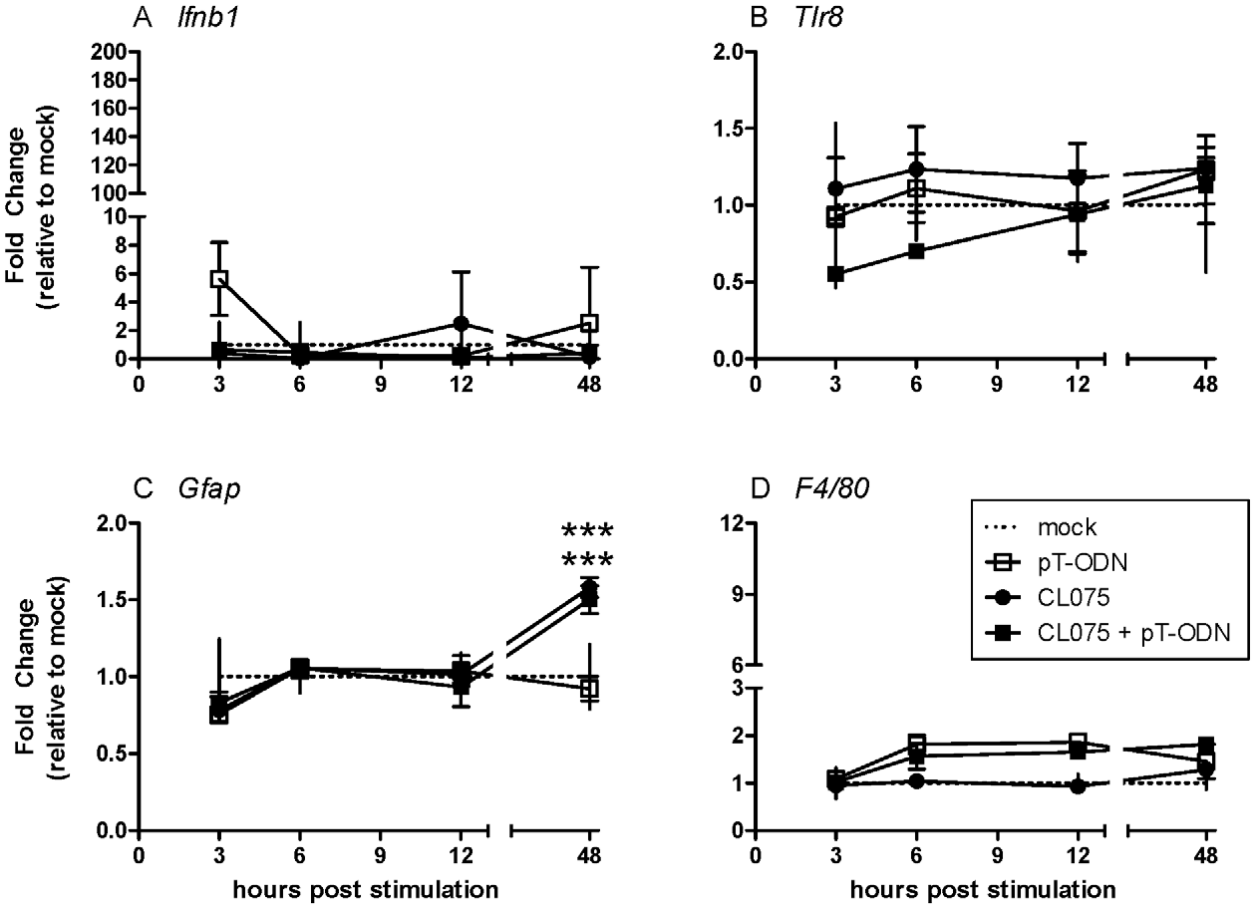
TLR7 is required for CL075/pT-ODN upregulation of cell activation markers and downregulation of *Tlr* mRNAs. Primary cortical cells from TLR7-deficient mice were cultured and stimulated as described in Fig. 2. RNA samples were processed for real-time quantitative RT-PCR analysis. Data were calculated as described in Fig. 2. Data are the mean ± SEM of three wells per group and are representative of two replicate experiments. Statistical analysis was completed by Two-way ANOVA with Newman-Keuls post test. Asterisks (*) directly above data symbols represent a statistically significant difference compared to media alone controls. *** P<0.001.

Not surprisingly, the cellular responses to CL075 that were not affected by pT-ODNs were also dependent on TLR7. This included the downregulation of *Tlr8* mRNA and the induction of *F4/80* mRNA (Fig. 5B–D). The morphological changes observed by CL075 or pT-ODN/CL075 treatment in Iba1 positive microglia (Fig.4 C, D) were not observed in cells from TLR7-deficient mice (Fig. 4E, F) indicating a necessity of TLR7 in CL075-induced microglia activation. However, the slight increase in *Gfap* mRNA was still observed in TLR7-deficient cultures (Fig. 5C). Therefore, CL075-induced glial activation was dependent on TLR7, with the exception of a low level of *Gfap* mRNA upregulation.

### pT-ODNs do not directly bind to TLR7/8 agonists, and do not affect internalization of the agonist

**Figure 6:**
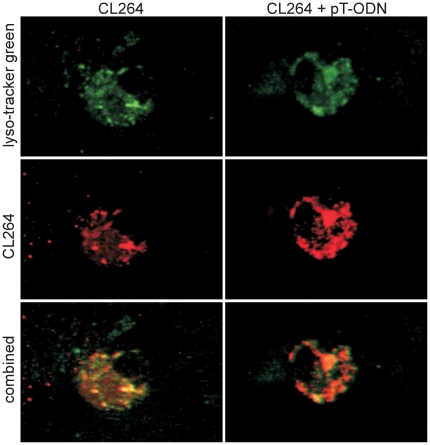
pT-ODNs do not alter endosomal localization of TLR7/8 agonists. Primary mixed cortical cells were grown on 8-chambered coverglass until semi-confluent. Cells were stimulated with 20 µM of rhodamine-labeled CL264 (left column) or 20 µM of rhodamine-labeled CL264 and 1 µM pT-ODN (right column) along with lyso-tracker green for 30 minutes. Each chamber was washed with media to remove unbound CL264 and analyzed by confocal microscopy. Images are representative of cells within the wells, with cells selected at random for imaging. Cells incubated with pT-ODNs alone or unstimulated cells did not have detectable rhodamine fluorescence (data not shown). Cells were followed for 3 h, with no significant differences noted in agonist localization.

**Figure 7:**
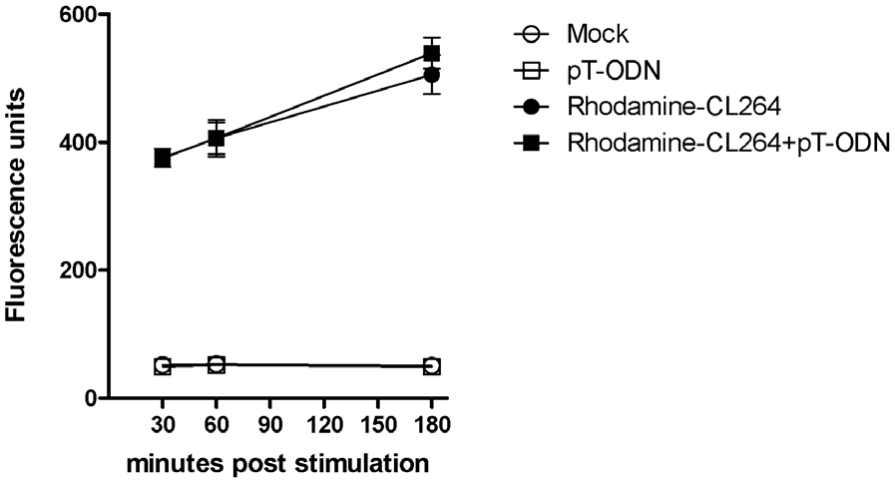
pT-ODNs do not alter uptake of TLR7/8 agonists. Primary cortical cells were grown in 24-well plates and stimulated with rhodamine-labeled CL264 in the presence or absence of 1 µM pT-ODN. At the indicated times, cells were washed 3X with PBS and fluorescence intensity measured (fluorescence units/ml/well). Data are the mean ± SEM of three wells per group and are representative of two replicate experiments. No significant differences were observed using a two-way ANOVA analysis between rhodamine-CL264 and rhodamine-CL264 with pT-ODN.

The experiments with TLR7 deficient mice indicate that pT-ODNs enhance the proinflammatory cytokine response to TLR7/8 agonists through stimulation of TLR7, even though pT-ODNs did not induce a strong innate immune response on their own. To examine the mechanism by which pT-ODNs could enhance TLR7/8 agonist stimulation, we examined whether pT-ODNs directly bound TLR7/8 agonists. We utilized rhodamine-labeled TLR7 agonist CL264, which induces similar stimulation to CL075, and examined whether pT-ODNs directly interacted with CL264 by measuring fluorescence polarization in the presence or absence of pT-ODNs. No difference in polarization was observed (data not shown). Confocal analysis using lyso-tracker green and rhodamine-labeled CL264 indicated no difference in cellular localization with the addition of pT-ODNs (Fig. 6). Similarly, analysis of cellular uptake of rhodamine-labeled CL264 demonstrated that the addition of pT-ODNs did not increase the amount of TLR7 agonist taken up by the cell (Fig. 7). Thus, pT-ODNs do not appear to affect TLR7/8 agonist uptake or internalization, indicating that the effect of pT-ODNs on cytokine responses appears to be due to enhanced TLR7 signaling within the cell.

### pT-ODNs induce a slight but detectable cytokine response in the absence of TLR7

**Figure 8:**
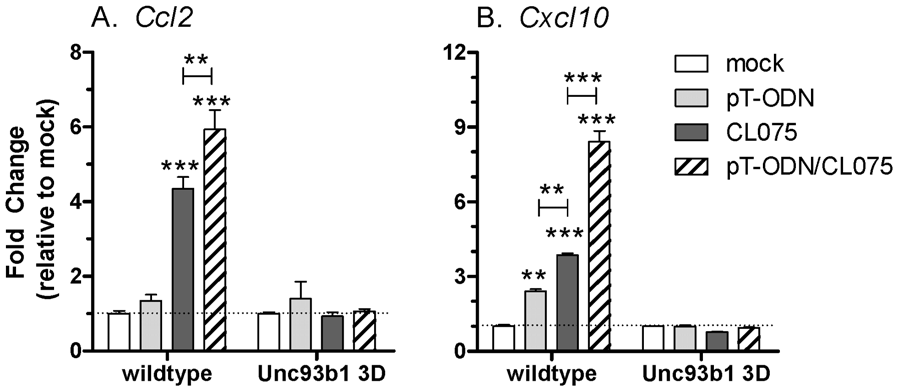
pT-ODN stimulation induction of low level CCL2 and CXCL10 production is dependent on Unc93b1. Primary cortical cells from wildtype C57BL/6 and C57BL/6 Unc93b1 3D mice were cultured and stimulated as described in Fig. 2. 5 µM of pT-ODNs, instead of 1 µM pT-ODN, was used in this study since this concentration induced optimal stimulation in cells from C57BL/6 mice in the presence or absence of 20 µM of CL075. Samples for RNA were collected at 6 hps and processed for real-time quantitative RT-PCR analysis. Data were calculated as described in Fig. 2. Data are the mean ± SEM of three wells per group and are representative of two replicate experiments. Statistical analysis was completed by one-way ANOVA with Newman-Keuls post test. Asterisks (*) directly above data symbols represent a statistically significant difference compared to media alone controls. Asterisks above lines between two columns indicate a statistically significant difference between the two indicated groups. ** P<0.01, *** P<0.001.

The above results indicate that the strong cytokine response induced by pT-ODN/CL075 stimulation was mediated by TLR7, not TLR8. However, in the absence of TLR7, a measurable, albeit minimal, increase in CCL2 and CXCL10 was observed following pT-ODNs stimulation alone in some, but not all, experiments (Fig. 3E, H, 8A–B, data not shown). This response suggested that pT-ODNs may be inducing a sub-optimal response that was not mediated via TLR7. To examine whether this sub-optimal response was mediated by endosomal TLRs, we utilized Unc93b1 3D mutant mice, which are deficient in transport of TLRs from the ER to the endosome. Interestingly, the cytokine response was completely absent in glial cells from Unc93b1 3D mice indicating that even the suboptimal cytokine response was dependent on TLR signaling (Fig. 8A–B). This response was not mediated by pT-ODN binding to TLR9 as TLR9 deficiency did not influence cytokine responses (data not shown). This response is also not likely to be mediated by TLR3, since ODNs do not induce TLR3 signaling. The dependency of the cytokine response on Unc93b1 was in contrast to 1.5 fold *Gfap* mRNA upregulation, which was still observed in Unc93b1 3D mice suggesting that the low level of *Gfap* upregulation was not an endosomal TLR-trigged mechanism. Thus, although the majority of the induction of innate immune responses by pT-ODNs in glial cells appears to be mediated via TLR7, there does appear to be a low, but detectable, cytokine response mediated by TLR8.

## Discussion

In the present study, we found that the TLR7/8 agonist CL075 induced the expression of glial cell activation markers and induced the production of multiple proinflammatory cytokines and chemokines. Addition of pT-ODN enhanced the expression of proinflammatory cytokines and chemokines. Both the CL075-induction of these responses and the enhancement of these responses by pT-ODN were dependent on TLR7. TLR7 agonists are currently being analyzed as potential therapeutics for the treatment of neurological maladies [27], [28]. However, the innate immune response to TLR7 agonists in the CNS is weak compared to other TLR ligands [19]. The ability of pT-ODNs to enhance TLR7/8 agonist-induced glial activation in this study suggests that pT-ODNs may be able to enhance the therapeutic potential of TLR7/8 agonists in the CNS.

The dependency of pT-ODN/CL075 stimulation on TLR7 in this study contrasts with previous reports where the addition of 13 to 20-mer pT-ODNs enhanced TLR7/8 agonist stimulation of murine TLR8, and suppressed TLR7/8 agonist stimulation of murine TLR7 in transfected HEK-293 cells [25], [29]–[31]. Peripheral blood mononuclear cells from TLR7-deficient mice also responded to pT-ODN/CL075 stimulation; however, this response was approximately 2 fold lower than the response in wildtype mice [25]. It is possible that murine TLR8 interacts more strongly with the adaptor molecule for TLR7/8, Myeloid Differentiation Factor 88 (MyD88), in human cells than in mouse cells and thus functions more efficiently in human cells than mouse cells to induce NFκB-related responses. Additionally, there may be adaptor or regulatory proteins expressed in astrocytes and microglia, but not in HEK cells that affect TLR8 signaling. It is also possible that the TLR8 transfected HEK cells express substantially higher levels of TLR8 than primary astrocytes and microglia and that the normal level of murine TLR8 expressed by glial cells is not sufficient for triggering detectable NFκB-related cytokine responses.

The current study did not rule out the possibility of the ability of TLR8 to induce a small level of cytokine production by glial cells in response to pT-ODN signaling. pT-ODNs induced a low level of CCL2 and CXCL10 in both wildtype and TLR7-deficient mice (Fig. 3). Additionally, a slight but insignificant increase in *Ifnb1* mRNA expression was also observed (Fig. 5A). These responses were not observed in glial cells derived from Unc93b1 3D mice indicating that an endosomal TLR was responsible for this low level response. The endosomal response is not likely to be due to TLR3 signaling since ODNs either do not induce TLR3 signaling and in some instances can even inhibit TLR3-mediated responses [32], [33]. Since the low-level induction of CCL2 and CXCL10 following pT-ODNs stimulation was still observed in TLR7- and TLR9- deficient mice, this cytokine response may be mediated by TLR8. Thus, murine TLR8 may be able to contribute to a neuroinflammatory response, although the significance of this response may be minimal compared to other TLR-mediated responses, which can induce pronounced glial activation.

TLR8 expression has also been detected on neurons in the developing mouse brain and stimulation of murine cortical neurons with the TLR7/8 agonist, R848, induced caspase 3 activation and inhibited dendrite outgrowth [34]. The signal transduction involved in TLR8-induced caspase 3 activation was not dependent on MyD88 or NFkB, suggesting an alternative pathway of signaling in neurons [35]. We did not detect any cell death in the mixed glia cultures, either with TLR7/8 agonists alone or with pT-ODN costimulation suggesting that TLR8 is not inducing cell death in glial cells.

The mechanism by which pT-ODNs enhances TLR7-induced cytokine responses in glial cells appears to be mediated at the intracellular level. Addition of pT-ODNs alone did not induce a significant cytokine response, with the exception of low level CCL2 and CXCL10 production (Fig. 3E, H). Thus, the increased cytokine response in co-stimulated cells does not appear to be due to additive or synergistic effects of signaling via another PRR. Furthermore, the complete ablation of this response in the absence of TLR7 indicates that the pT-ODN response is not due to a synergistic effect between two different PRRs. The addition of pT-ODNs did not enhance cellular uptake of TLR7/8 agonists and did not alter the overall amount of agonist in the cell over time (Fig. 7). Furthermore, no difference was found in agonist localization to the endosome (Fig. 6). Thus, the mechanism by which pT-ODNs influence TLR7/8 agonist binding appears to be at the level of agonist/receptor interaction. pT-ODNs may alter the environment in the endosome to create a more favorable environment for TLR7/8 agonist binding to TLR7 or they may directly interact with either the TLR7/8 agonist or the receptor inside the cell. It is also possible that pT-ODNs may stimulate cells to shuttle TLR7 from the endoplasmic reticulum to the endosome where it can interact more readily with TLR7/8 agonists. The ability of pT-ODNs to act in synergy with TLR7/8 agonists to induce strong TLR7-dependent cytokine production in glia cells suggest that the combination of these ligands would result in heightened neuroinflammatory responses in the CNS. Thus, pT-ODNs may be useful in potentially enhancing the adjuvant/therapuetic properities of TLR7/8 agonists in the CNS and other tissues.

## Materials and Methods

### Mice

TLR7-deficient C57BL/6 mice [13] were kindly provided by S. Akira (Osaka University, Osaka, Japan) and were backcrossed with Inbred Rocky Mountain White (IRW) mice for at least 10 generations [19]. IRW mice and TLR7-deficient IRW mice were used for the present study. Mice were genotyped to confirm TLR7 knockout allele as previously described [17], [19]. Unc93b1 3D mice were obtained from the Mutant Mouse Regional Resource Center at University of California, Davis. All of the animal procedures were approved by and conducted in accordance with the Louisiana State University Animal Care and Use Committee guidelines or the Rocky Mountain Laboratories Animal Care and Use Committee guidelines under protocols LSU06-120 and RML2008-46.

### Primary cortical cultures

Primary mixed cortical cultures were generated from the cortical tissue of 1- to 2-day-old neonatal mice. Intact brains were removed and dissected free of meninges. The midbrain and cerebellum were removed and the cortices were placed in 2% glucose/PBS and gently triturated using a 10 ml pipet. Cells were pelleted at 244 g for 5 min, and a single-cell suspension was made by triturating with a 1-ml syringe and 20 gauge needle. Cells were cultured in 25-cm^2^ Primaria tissue culture flasks (BD Bioscience) in Dulbecco's modified Eagle's medium (DMEM) containing 10% fetal bovine serum (FBS), 100 units penicillin and streptomycin and maintained at 37°C, 5% CO_2_. When cultures reached 90 to 95% confluency in the flask, they were treated with 0.25% Trypsin, 1 mM EDTA in HBSS (0.25% Trypsin-EDTA, Gibco) for 5 minutes, harvested and replated in 12-well plates for agonist stimulation or 6-well plates for flow cytometry analysis. Immunohistochemical analysis of these cultures demonstrated that the primary cell type was astroglia, but also contained a number of microglia. Detection of neurons and other cells was rare.

### Astrocyte and microglia cultures

For the generation of astrocyte cultures and for analyzing TLR7 and TLR8 expression in astrocytes and microglia, single cell suspensions were generated as described above. However, following trituration, cells were suspended in 2 ml of 70% percoll and transferred to the bottom of 30%, 0% step percoll gradient. The gradients were centrifuged at 500 g for 20 min. The microglia cell population was collected between 30% and 70% percoll layers, washed in PBS, and seeded at 5×10^5^ in Primaria T-25 flasks containing DMEM with 10% FBS and 20% LADMAC culture supernatant (mouse bone marrow cells producing macrophage colony stimulating factor/M-CSF). The astrocyte rich cell population was collected between 0% and 30% percoll layers, washed in PBS, and seeded at 2×10^5^ cells in Primaria T-25 flasks containing DMEM with 10% FBS. When cells reached confluency (7–10 days), flasks containing astrocyte rich cells (0/30 fraction) were orbitally shaken overnight at 250 RPM to remove any remaining microglia as well as any oligodendrocytes. Astrocytes were removed from the flasks by treating with 0.25% Trypsin-EDTA (Gibco), and microglia were removed from confluent T-25 flasks using a cell scraper for use in flow cytometric analysis. Astrocyte cultures were greater than 95% GFAP positive and microglia were greater than 95% F4/80 and Iba1 positive.

### Cell Stimulation

When primary cortical cultures were approximately 80% confluent, they were used for agonist stimulation. The TLR7/8 agonist CL075, a thiazoloquinoline compound also known as 3M002 (Invivogen, San Diego, CA) was prepared in endotoxin-free water, aliquoted at stock concentrations of 20 mM and was thawed only once prior to use. A 20-mer of pT-ODN (Invitrogen) was prepared in endotoxin-free water, aliquoted at stock concentrations of 2 mM and was thawed only once prior to use. Cultures were stimulated with optimal concentrations of 20 µM of CL075, and 1–5 µM of pT-ODN depending on experiment. In costimulation experiments, 1 µM was optimal in cultures generated from IRW mice, whereas 5 µM was optimal in cultures from C57BL/6 mice.

### Flow Cytometry

Cells were analyzed for TLR7 and TLR8 protein expression by intracellular staining. Cells were fixed for 20 min in 2% paraformaldehyde, permeabilized with 0.1% saponin in PBS (pH 7.0), and then incubated with rat anti-mouse CD16/CD32 antibodies (BD Biosciences) to block non-specific antibody binding to Fc receptors. Cells were then incubated with polyclonal rabbit anti-TLR7 antibody (Zymed), goat anti-TLR8 antibody (Capralogics), polyclonal rabbit anti-glial fibrillary acidic protein (GFAP) antibody (Dako), mouse anti-F4/80 antibody (eBioscience), rabbit anti-Iba1 antibody (Wako Inc.) or isotype control antibody overnight at 4°C. The next day, cells were incubated with the relative secondary antibody conjugated to AlexaFluor 488 (Invitrogen) in 0.1% saponin/PBS. Cells were washed twice with PBS, resuspended in 3% BSA in PBS, and analyzed on a FACSAria flow cytometer (BD Biosciences) using FACSDiva software (BD Biosciences). Data analyses were performed using FCS3 Express software (De Novo). GFAP, F4/80 and Iba1 antibodies were used to confirm cell purity.

### Immunocytochemistry Analysis

Primary cortical cultures in 8-chamber culture slides (BD Biosciences) at 70–80% confluency were used for immunocytochemical analysis. Cells were fixed in 4% para-formaldehyde for 15 min, permeabilized with 0.1% Triton X-100 and 0.1% Sodium citrate for 30 min, and blocked using normal donkey serum blocking solution (PBS containing 2% donkey serum, 1% BSA, 0.1% cold fish skin gelatin, 0.1% Triton X-100, and 0.05% Tween 20) for 30 min. Cells were then incubated overnight at 4°C with the appropriate concentration of GFAP, Iba1 or F4/80 in the normal donkey serum blocking solution. Cells were stained using antibody concentrations stated above for flow cytometric analysis. Cells were then incubated with the relative secondary antibody conjugated to AlexaFluor 488 (Invitrogen). The slides were covered with a glass coverslip using Fluoro-Gel II with DAPI mounting solution (Electron Microscopy Sciences). Images were pseudocolored and overlaid using Olympus MicroSuite FIVE or Nikon Elements NIS Basic Research software.

### Analysis of mRNA Expression by Real-time PCR

Total RNA was extracted from primary cell cultures at 48 hps using an RNA isolation kit (Zymo Research) following manufacturer's instructions. RNA was treated with DNaseI for 30 minutes and re-purified using an RNA clean-up kit (Zymo Research). cDNA was generated from RNA samples using an iScript reverse transcription kit (Bio-Rad) following manufacturer's instructions. cDNA was diluted 5 fold in RNase-free water prior to use in real-time PCR. Primers for real-time PCR analysis are shown in Table 1. All primers used for real-time PCR analysis were designed using Primer3 software [36]. Primer sequences were blasted against the National Center for Biotechnology Information (NCBI) database to confirm that all primer pairs were specific for the gene of interest and that no homology to other genes was present. PCR reactions were prepared using SYBR green mix with Rox (Bio-Rad) in a 10 µl volume with approximately 10 ng of cDNA and 1.8 µM forward and reverse primers. Samples were run in triplicate on an ABI PRISM 7900 Sequence Detection System (Applied Biosystems). Analysis of dissociation curves was used to confirm the amplification of a single product for each primer pair per sample. Confirmation of a lack of DNA contamination was achieved by analyzing samples that had not undergone reverse transcription. Untranscribed controls had at least a 1,000 fold lower expression level than analyzed samples or were negative for all genes after 40 cycles. Gene expression was quantified by the cycle number at which each sample reached a fixed fluorescence threshold (CT). To control for variations in RNA amounts among samples, data were calculated as the difference in CT values (log2) between the housekeeping gene, *Gapdh,* and the gene of interest for each sample (ΔCT  =  CT *Gapdh* − CT gene of interest). Data were calculated as a percentage of *Gapdh* expression for each gene of interest per sample. These data were then calculated as fold expression relative to the average of mock samples for each gene and each group.

**Table 1:** Primers used for real-time RT-PCR analysis.

Common Name	NCBI Gene Symboland Identification No.	Forward Primer	Reverse Primer
Monocyte chemoattractant protein 1 (MCP-1)	*Ccl2*: 20296	TCCCAATGAGTAGGCTGGAG	CCTCTCTCTTGAGCTTGGTGA
Inducible protein 10 (IP-10)	*Cxcl10*: 15945	CAGTGAGAATGAGGGCCATAGG	CTCAACACGTGGGCAGGAT
F4/80	*Emr1*: 13733	TTACGATGGAATTCTCCTTGTATATCA	CACAGCAGGAAGGTGGCTATG
Glyceraldehyde-3-phosphate dehydrogenase	*Gapdh*: 407972	TGCACCACCAACTGCTTAGC	TGGATGCAGGGATGATGTTC
Glial fibrillary acidic protein	*Gfap*: 14580	CGTTTCTCCTTGTCTCGAATGAC	TCGCCCGTGTCTCCTTGA
G-coupled protein 84	*Gpr84*: 80910	CTGACTGCCCCTCAAAAGAC	GGAGAAGTTGGCATCTGAGC
Interferon beta1	*Ifnb1*: 15977	AGCACTGGGTGGAATGAGAC	TCCCACGTCAATCTTTCCTC
Toll-like receptor 7	*Tlr7*: 170743	GGCATTCCCACTAACACCAC	TTGGACCCCAGTAGAACAGG
Toll-like receptor 8	*Tlr8*: 170744	ACAATGCTCCATTTCCTTGC	CTGAGGGAAGTGCTGGAAAG
Tumor necrosis factor	*Tnf*: 21926	CCACCACGCTCTTCTGTCTAC	GAGGGTCTGGGCCATAGAA

### Analysis of Cytokine And Chemokine Protein Expression by Multiplex Bead Array

At 48 hps, supernatants from primary cortical cultures were collected and stored at −80°C. Just before use, supernatants were thawed to room temperature. Supernatants were analyzed for cytokine and chemokine proteins using a 20-plex multiplex bead array (BioSource) on a Luminex 100 instrument (Bio-Rad) following manufacturer's instructions. The cytokines analyzed were CCL2 (MCP-1), CCL3 (MIP-1α), CXCL9 (MIG), CXCL10 (IP-10), fibroblast growth factor (FGF), granulocyte monocyte colony stimulation factor (GM-CSF), interferon gamma (IFN-γ), Interleukin-1α (IL-1 α), IL-1 β, IL-2, IL-4, IL-5, IL-6, IL-10, IL-12p40, IL-13, IL-17, CXCL1 (KC), TNF, and vascular endothelial growth factor (VEGF). Data were calculated as pg/ml using a standard curve from in-plate standards. CCL2 (MCP1), IFNα and IFNβ protein levels in culture supernatants were measured using cytokine-specific ELISA assays (R&D Systems, Minneapolis, MN) following manufacturer's instructions.

### Agonist uptake assay

Glial cells were grown in 96-well plates until over 80% confluent. Cells were stimulated with mock, 1 µM pT-ODN, 20 µM rhodamine-labeled CL264 or 20 µM rhodamine-labeled CL264 and 1 µM pT-ODN. Cells were then incubated at 37°C and 5% CO_2_ for 30 min, 1 hr or 3 hr. At each time point, cells were washed three times with PBS and analyzed for rhodamine uptake. Cells were then lysed in lysis buffer (0.5% Triton X-100, 0.5% sodium deoxycholate, 150 mM NaCl, 50 mM Tris HCl, pH 7.4 and 8 mM EDTA) to release fluorescence into solution, and the fluorescence intensity was quantitated using a microplate reader (Polar Star Omega, BMG Labtech).

### Cellular localization assay

For confocal microscopy analysis, glial cells were grown on Lab-Tek® II Chamber # 1.5 coverglasses and then stimulated with mock, 1 µM pT-ODN, 20 µM rhodamine-labeled CL264 or 20 µM-rhodamine labeled CL264 combined with 1 µM pT-ODN. Lyso-tracker green at 5 µM was added to each well at the same time. After 30 min incubation at 37°C and 5% CO_2_, cells were washed with fresh media two times and kept in 0.1 M NH_4_Cl in media. All images were taken using a Zeiss 510 Meta Confocal Microscope.

### Statistical Analysis

All of the statistical analyses were performed using Graph Pad Prism software (San Diego, CA) using the appropriate statistical test as described in the figure legends.
